# Mutations in the heparan sulfate backbone elongating enzymes EXT1 and EXT2 have no major effect on endothelial glycocalyx and the glomerular filtration barrier

**DOI:** 10.1007/s00438-022-01854-w

**Published:** 2022-02-01

**Authors:** Ramzi Khalil, Margien G. S. Boels, PALGA Group, A. Bezuijen, J. E. Boers, P. C. de Bruin, M. A. A. M. van Dijk, P. Drillenburg, A. F. Hamel, H. M. Hazelbag, G. N. Jonges, R. E. Kibbelaar, K. H. Lam, H. van der Linden, J. van Marsdijk, C. Meijer, I. D. Nagtegaal, J. J. Oudejans, J. J. T. H. Roelofs, L. Rozendaal, S. H. Sastrowijoto, M. M. Smits, J. Stavast, Bernard M. van den Berg, Jan A. Bruijn, Ton J. Rabelink, Pancras C. W. Hogendoorn, Hans J. Baelde

**Affiliations:** 1 Department of Pathology, Leiden University Medical Center, L1Q, Room P0-107, P.O. Box 9600, 2300 RC Leiden, The Netherlands; 2 The Einthoven Laboratory for Vascular and Regenerative Medicine, Department of Internal Medicine, Division of Nephrology, Leiden University Medical Center, Leiden, The Netherlands

**Keywords:** Multiple osteochondromas, Hereditary multiple exostosis, Endothelium, Albuminuria, Glomerular filtration barrier, Glycocalyx, Proteinuria, Bone neoplasm

## Abstract

In this study, the effect of heterozygous germline mutations in the heparan sulfate (HS) glycosaminoglycan chain co-polymerases EXT1 and EXT2 on glomerular barrier function and the endothelial glycocalyx in humans is investigated. Heparan sulfate (HS) glycosaminoglycans are deemed essential to the glomerular filtration barrier, including the glomerular endothelial glycocalyx. Animal studies have shown that loss of HS results in a thinner glycocalyx. Also, decreased glomerular HS expression is observed in various proteinuric renal diseases in humans. A case report of a patient with an *EXT1* mutation indicated that this could result in a specific renal phenotype. This patient suffered from multiple osteochondromas, an autosomal dominant disease caused by mono-allelic germline mutations in the *EXT1* or *EXT2* gene. These studies imply that HS is indeed essential to the glomerular filtration barrier. However, loss of HS did not lead to proteinuria in various animal models. We demonstrate that multiple osteochondroma patients do not have more microalbuminuria or altered glycocalyx properties compared to age-matched controls (*n* = 19). A search for all Dutch patients registered with both osteochondroma and kidney biopsy (*n* = 39) showed that an *EXT1* or *EXT2* mutation does not necessarily lead to specific glomerular morphological phenotypic changes. In conclusion, this study shows that a heterozygous mutation in the HS backbone elongating enzymes EXT1 and EXT2 in humans does not result in (micro)albuminuria, a specific renal phenotype or changes to the endothelial glycocalyx, adding to the growing knowledge on the role of EXT1 and EXT2 genes in pathophysiology.

## Background

The effect of heterozygous germline mutations in *EXT1* or *EXT2* on the glomerular filtration barrier, with special attention to the endothelial glycocalyx, is investigated in this study. We hypothesized that patients with a mutation in *EXT1* or *EXT2* have a perturbed endothelial glycocalyx and glomerular basement membrane that results in changes in renal morphology and altered glomerular permeability. Therefore, we performed a cross-sectional observational study in a cohort of patients with *EXT1* and *EXT2* mutations to investigate endothelial glycocalyx health and the glomerular barrier function in humans. In addition, we identified a historic cohort of renal biopsy or autopsy tissue from patients with osteochondromas in which we examined whether a specific renal morphological phenotype is present. HS is made up of repeating disaccharide units of *N*-acetyl glucosamine and glucuronic acid, covalently attached to a core protein, such as syndecans and glypicans (Esko and Selleck 2002). Biosynthesis of HS comprises three steps: chain initiation, chain elongation, (requiring the EXT1 and EXT2 co-polymerase), and chain modification. Depending on the modification state of HS, various sulfate groups are attached to the chain and thus infer negative charge, anti-coagulatory properties, and the capability to bind various cytokines and chemokines (Xu and Esko 2014).

HS has been studied extensively in the glomerular filtration barrier (GFB) (Kanwar and Farquhar 1979; Kanwar et al. 1980). The GFB consists of fenestrated endothelial cells covered by glycocalyx, the glomerular basement membrane, and specialized visceral epithelial cells (podocytes) with interdigitating foot processes. There has been a longstanding discussion on whether HS is essential to glomerular permeability (Garsen et al. 2014). Glomeruli of patients with lupus nephritis, membranous glomerulonephritis, minimal change disease, and diabetic nephropathy showed a decreased staining of heparan sulfate (Born et al. 1993). Degradation of HS in the glycocalyx by heparanase increases glomerular permeability to albumin, results in increased accessibility to autoantibodies, and loss of HS was observed in various proteinuric renal diseases (Born et al. 1993; Singh et al. 2007; Hogendoorn et al. 1988). On the other hand, a genetic HS deficiency has been shown not to result in significant proteinuria in various experimental animal models (Chen et al. 2008; Harvey et al. 2007; Goldberg et al. 2009; Sugar et al. 2014; Khalil et al. 2019).

Gleadle et al*.* reported on a patient with a mono-allelic germline mutation in an HS polymerizing gene, *EXT1*, that presented with a nephrotic syndrome. Histology from a renal biopsy of this patient showed specific changes on EM of fibrillar deposition in the GBM and mesangium (Roberts and Gleadle 2008). Based on this case, the renal disease ‘glomerulopathy of hereditary multiple exostoses’ is recognized as a separate disease entity (Colvin and Chang 2015). *EXT1* or *EXT2* loss of function mutations results in the autosomal dominant disease multiple osteochondromas (MO, OMIM No. 133700 and 133,701), previously also known as hereditary multiple exostoses (HME) and multiple hereditary multiple exostoses (MHE) (Wuyts and Hul 2000). MO is defined as a disease ‘characterized by development of two or more cartilage capped bony outgrowths (osteochondromas) of the long bones.’ (Bovee 2008; Bovee and Hogendoorn PC: Multiple osteochondromas. In, 2002). More than 85% of MO patients have a germline mutation in the *EXT1* or *EXT2* gene (Bovee 2008). The *EXT1* and *EXT2* gene products form a co-polymerase that initiates chain elongation in heparan sulfate glycosaminoglycan synthesis (Esko and Selleck 2002). Existence of another exostosin gene, *EXT3,* has been suggested but not proven (Merrer et al. 1994). However, as stated above, the diagnosis is often based on radiological and clinical assessment. Therefore, in many patients, genetic testing is regarded as optional in establishing the diagnosis (Hameetman et al. 2004).

In MO patients, a decrease in HS levels has been described in the circulatory system and in exostosis growth plates (Anower et al. 2013; Hecht et al. 2002). However, the effect of heterozygous *EXT1* and *EXT2* mutations on the endothelial glycocalyx have not yet been described. Moreover, it is currently unknown whether a loss of HS as a result of perturbed HS assembly results in an increase in glomerular permeability in humans.

HS is also a major structural component of the glycocalyx, a coat of sugars, proteins, and lipids, lining the luminal side of the endothelium (Reitsma et al. 2007). Endothelial dysfunction is an important process in both cardiovascular and renal disease (Deanfield et al. 2007; Stam et al. 2006). Recent technological advances have now made it possible to assess the red blood cell (RBC) count accessibility to the endothelial surface glycocalyx, in which a healthy glycocalyx is reflected by a low perfused boundary region (PBR) and a glycocalyx at risk is reflected by a high PBR (Dane et al. 2014; Lee et al. 2014).

## Methods

### Ethics

Ethical approval for this study was obtained through the LUMC Medical Ethical Committee under registration number P15.106. Anonymized patient material from the PALGA search was handled in accordance with institutional guidelines, Good Research Practice, and the Code of conduct for responsible use.

### MO patient study population

Patients were approached through the Dutch HME-MO Association (www.hme-mo.nl). Controls were obtained from the general population and matched for age and gender. All participants were required to be capacitated and over 18 years of age. After obtaining informed consent, participants were handed a digital questionnaire. Data on mutation status, use of medication, smoking, hypertension, and relevant medical history were collected. Participants were asked to perform one site visit, during which a glycocalyx measurement was performed and urine samples were collected.

### Glycocalyx measurements

Sublingual microvasculature was visualized non-invasively using a sidestream dark field MicroScan Video Microscope (MicroVision Medical, Inc., Wallingford, PA), connected to Glycocheck™ acquisition and analysis software (Microvascular Health Solutions Inc., Salt Lake City, UT, USA) to automatically acquire microvascular video recordings after predefined image quality criteria (motion, intensity, and focus) are fulfilled. Each complete measurement consists of at least ten 2-s videos (40 frames/video), containing a total of about 3000 vascular segments of 10 μm length each. During the measurement, the camera is moved to different positions under the tongue to obtain different sublingual microvascular sites. The vascular segments are automatically subjected to a quality check by the GlycoCheck™ software, discarding invalid segments. Next, the software obtains up to 840 radial intensity profiles for each valid vascular segment, and based on the RBC column width (RBCW) it automatically groups vessels from 4 to 25 μm diameter in 22 separate diameter classes (1 μm each). Data from two complete measurements were extracted, analyzed, and averaged offline to avoid sampling error and to counterbalance spatial–temporal heterogeneity of the sublingual microcirculation. These analyses resulted in the following parameters: perfused capillary density; capillary blood volume; and dynamic perfused boundary region (PBR). PBR is a measure for glycocalyx quality by measuring dynamic lateral RBC movement into the glycocalyx: a larger PBR reflects a disturbance of the glycocalyx (Dane et al. 2014; Lee et al. 2014).

Detailed information about the video acquisition and software used is described elsewhere (Lee et al. 2014).

### Urinary albumin/protein excretion

Spot urine samples were collected during the site visit and analyzed for albumin, total protein, and creatinine at the LUMC department of Clinical Chemistry. Albumin-creatinine ratio (ACR) and protein-creatinine ratio (PCR) were used to identify the presence of (micro)albuminuria and (micro)proteinuria according to the KDIGO CKD guideline (Group CW 2013).

### Historic PALGA cohort selection

The Dutch pathology national automated archive (PALGA) was searched for cases containing both the search terms ‘kidney and biopsy’ and ‘osteochondroma and multiple osteochondromas,’ to identify potential MO patients who have undergone a renal biopsy or autopsy in which renal tissue was collected between 1978 and 2014. The slides and EM material of the identified cases were requested from the corresponding hospitals. Sections were assessed for specific light microscopic changes and, if available, irregularities on EM.

### Statistical analyses

Baseline characteristics are expressed as mean (± standard deviation [SD]), or as percentage. Groups were matched for age and gender. Difference between groups in sublingual measures and albuminuria was determined using unpaired t-tests. ANOVA was used for side-by-side comparison of PBR in various vessel sizes. Pearson’s correlation coefficient was calculated to assess correlations. Graphpad Prism (version 8.4.2 (GraphPad Software, San Diego, CA, USA) was used for statistical analyses. *p* < 0.05 was considered statistically significant.

## Results

### Patient characteristics

Twenty MO patients responded to the call for our study. 1 patient retracted from the study before performing the site visit. Of the 19 remaining patients, 6 had a confirmed *EXT1* (4) or *EXT2* (2) mutation and 13 had a clinical diagnosis of MO without mutational confirmation (Hameetman et al. 2004). 27 controls, of which 19 are age and sex matched, were selected from the general population. No differences were observed in gender, age, or body mass index (Table 1).

**Table 1:** Patient characteristics

	MO patients	Healthy controls
Subjects	19	19
Age, mean (SD), y	45 (± 15.6)	45 (± 16.0)
Men, *n* (%)	2 (10.5)	2 (10.5)
BMI (SD)	26,0 (± 6.5)	23.4 (± 2.8)
Smoking, *n* (%)	1 (5.3)	1 (5.3)
Hypertension, *n* (%)	4 (21.1)	0
ACE inhibitor, *n* (%)	2 (10.5)	0
ARB, *n* (%)	0	0
NSAID use, *n* (&)	7 (33.3)	3 (14.3)
Diabetes, *n* (%)	2 (10.5)	0
Type 1, *n* (%)	0	0
Type 2, *n* (%)	2 (10.5)	0
EXT1 mutation, *n* (%)	4 (21.1)	N/A
EXT2 mutation, *n* (%)	2 (10.5)	N/A
Mutation unknown, *n* (%)	13 (68.4)	N/A

*BMI* Body Mass Index, *ACE* angiotensin converting enzyme, *ARB* angiotensin 2 receptor blocker, *NSAID* non-steroidal anti-inflammatory drugs, *EXT* exostosin

### *EXT1/EXT2* mutations do not result in changed microvascular endothelial glycocalyx properties

**Fig. 1:**
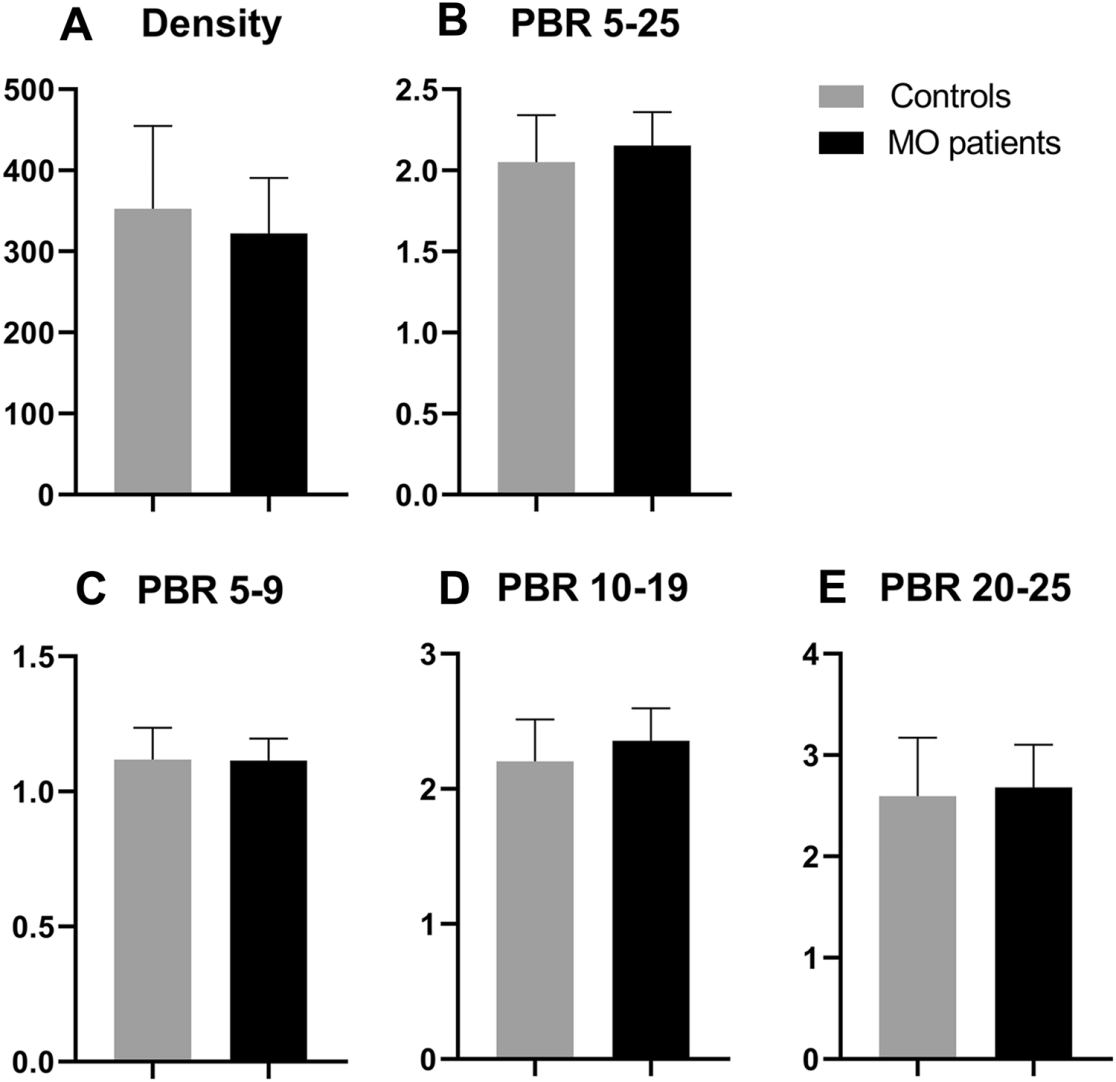
MO patients have normal vascular density and perfused boundary region. **A** through **E** Summary of vascular density (**A**) and perfused boundary region (**B** through **E**). No difference is found in both vascular density and PBR between controls and MO patients. Subanalysis of PBR in vessels of 5–9 µm (**C**), 10–19 µm (**D**), and 20–25 µm (**E**) also shows no difference between controls and MO patients. *p > 0.99

Since mutations in one of the HS elongation enzymes *EXT1* and *EXT2* could result in compositional changes of the endothelial glycocalyx, we investigated possible perturbations in glycocalyx properties. Sublingual measurements of the microcirculation of control and MO patients did not reveal a difference in perfused vascular density (352.69 ± 102.16 vs. 322.25 ± 68.17 µm/mm^2^, respectively; *p* > 0.99, Fig. 1A) nor in changes in the perfused boundary regions (2.05 ± 0.29 vs. 2.15 ± 0.21 µm, respectively; *p* > 0.99, Fig. 1B). Subanalysis of PBR in vessels 5–9 µm, 10–19 µm, and 20–25 µm also showed no difference between MO patients and controls (*p* > 0.99 in all instances Fig. 1C through E). No correlation was found between increasing age and any of the used outcome parameters, which were density, PBR 5–25, PBR 5–9, PBR 10–19, and PBR 20–25. This was the case in the overall group (*r* = 0.20, 0.21, − 0.13, 0.20, and 0.24 with *p* = 0.19, 0.18, 0.42, 0.21, and 0.13, respectively) as well as when analyzing the MO patient (*r* = 0.28, − 0.12, − 0.08, − 0.25, and 0.06 with *p* = 0.27, 0.66, 0.77, 0.34, and 0.82, respectively) and control group (*r* = 0.18, 0.35, − 0.17, 0.38, and 0.33 with *p* = 0.37, 0.08, 0.43, 0.06, and 0.11, respectively) separately.

### *EXT1/EXT2* mutations do not directly induce proteinuria

**Fig. 2:**
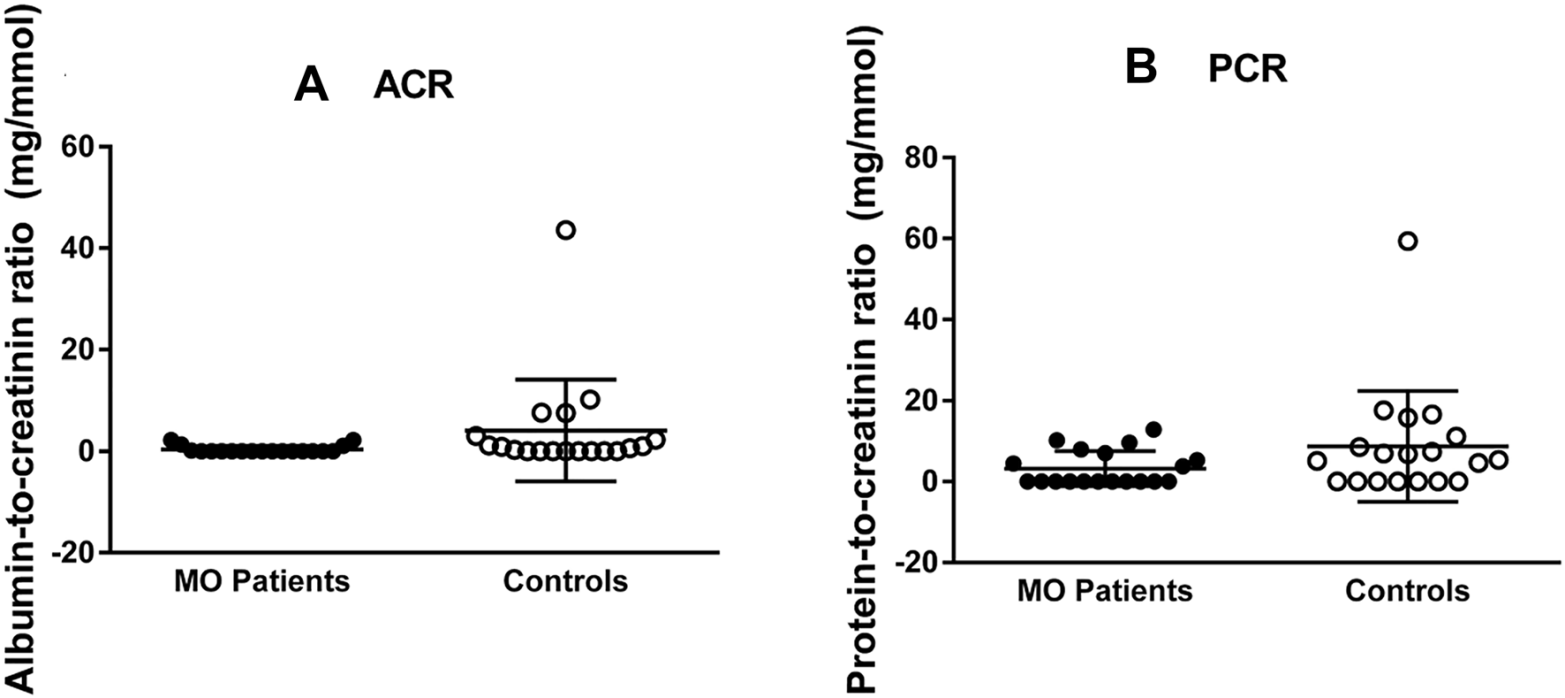
MO patients are normoalbuminuric. MO patients do not have a higher albumin-to-creatinine (**A**) nor a higher protein-to- creatinine ratio (B). *p* = 0.1251 and *p* = 0.1282, respectively

Analysis of the collected urine samples was performed by matching MO patients to controls based on age and gender. Analysis revealed no difference in ACR nor PCR of patients compared to controls (*p* = 0.13, Fig. 2). All MO patients in this study were found to be normoalbuminuric. Five control cases were microalbuminuric and one control case was albuminuric according to the KDIGO CKD guideline (Group CW 2013). No correlation was found between age and ACR or PCR in both patients (*r* = − 0.09 and 0.10 with *p* = 0.72 and 0.69*)* and controls (*r* = − 0.24 and − 0.19 with *p* = 0.33 and 0.43, respectively).

### Renal morphological change in patients with osteochondromas

Searching the Dutch pathological anatomy national automated archive (PALGA) for cases in which both ‘osteochondroma’ and ‘kidney’ are mentioned, resulted in 178 reports from 63 patients. After excluding cases in which no renal tissue was present, slides and EM material from 77 reports out of 39 cases were requested from the corresponding hospitals. We received material from 24 cases. Out of these cases, at least 4 can be classified as having MO based on clinical criteria available in the pathology reports. Genetic data of this cohort are unknown.

**Fig. 3:**
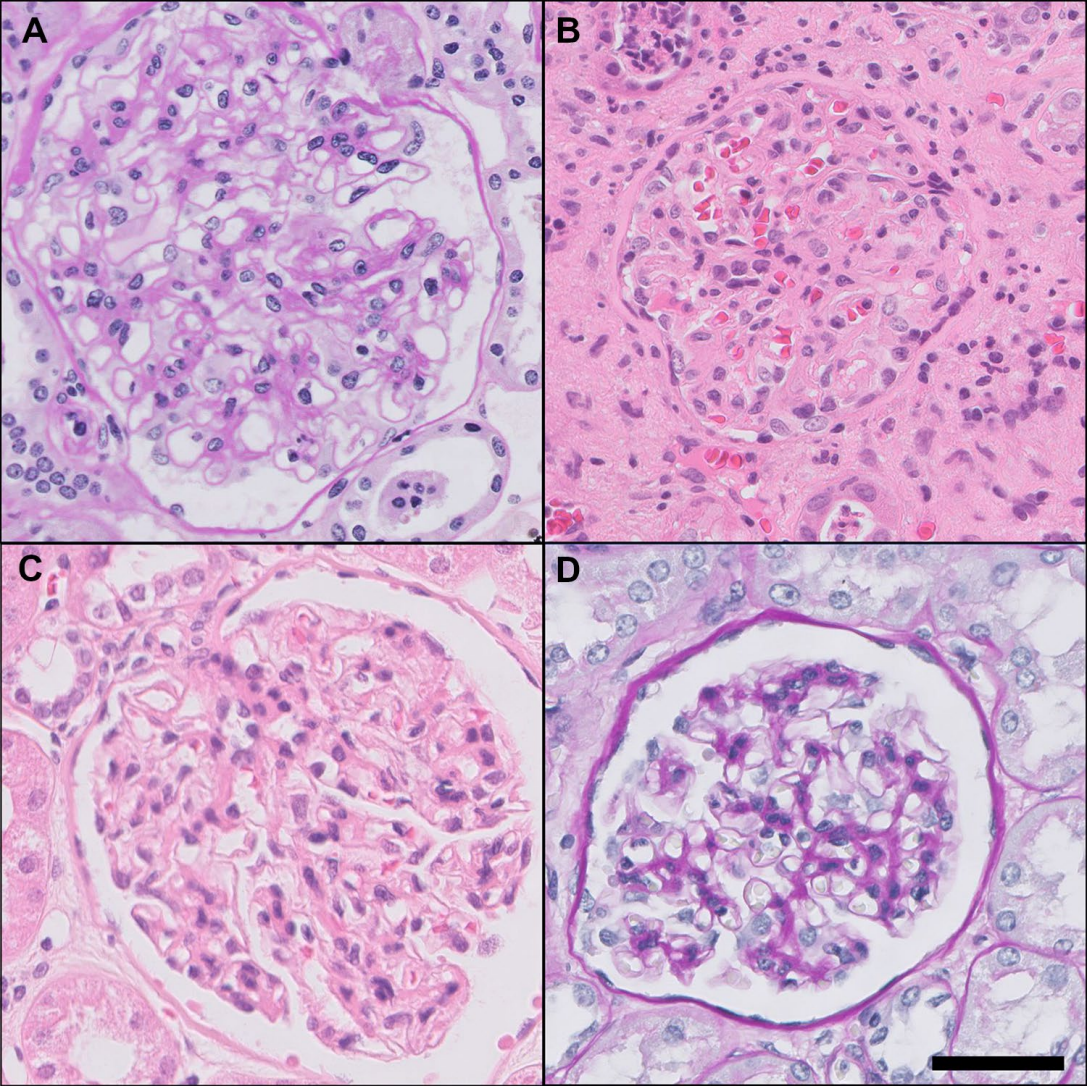
Light microscopy of glomeruli from MO patients shows relatively normal morphology. Snapshots of glomeruli from MO patients are shown. No specific lesions other than those related to the primary disease were observed. The examples are derived from patients with IgA nephropathy (**A**, PAS), glomerulonephritis after transplantation (**B**, H&E), and neoplastic diseases (**C**, H&E and **D**, PASD). Original magnification 40x. Scale bar = 50 µm

**Fig. 4:**
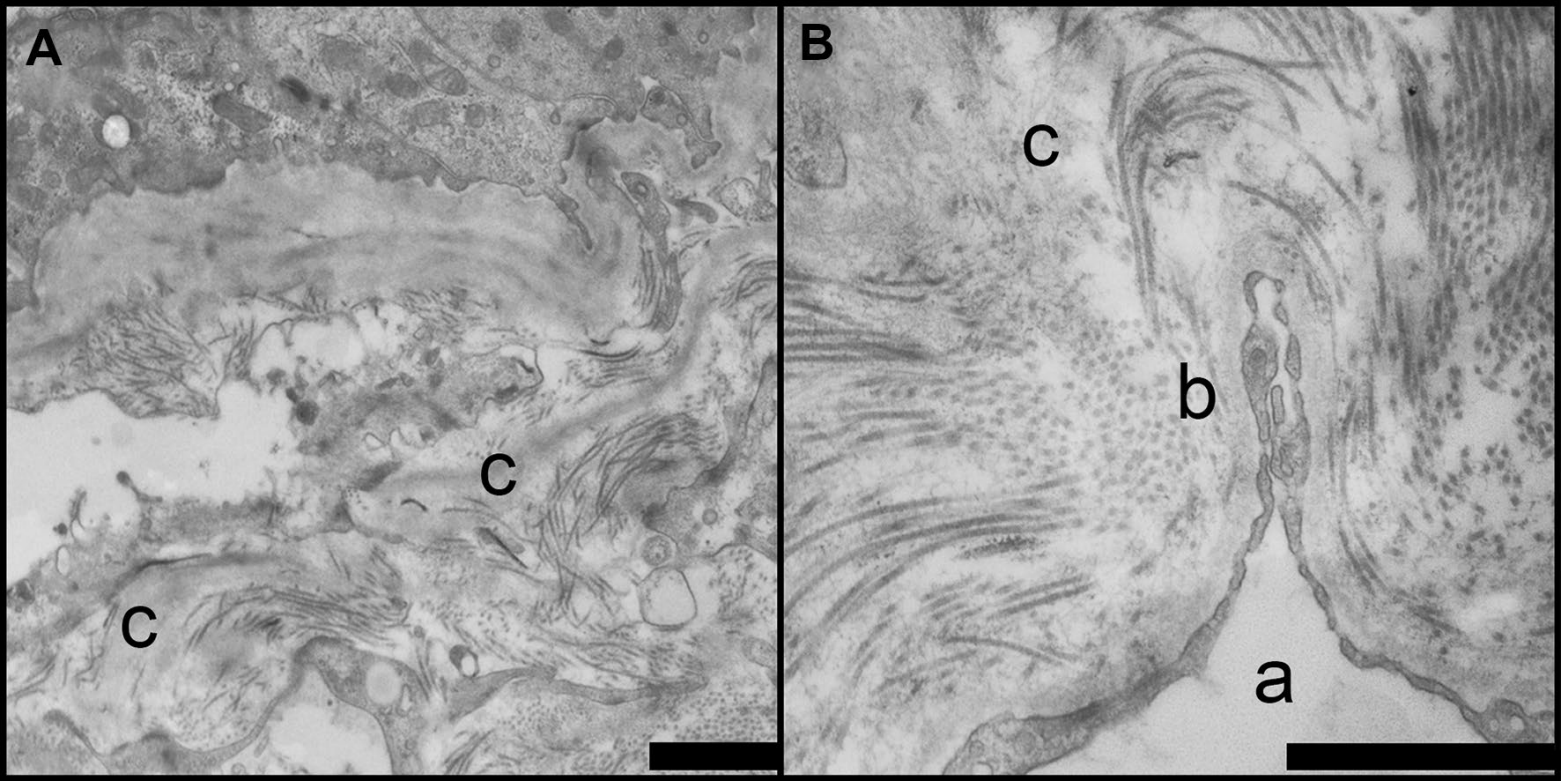
Glomerular fibril deposition HME-MO glomerulopathy. **A** and **B** Deposition of large fibrils with a diameter of 140 nm was observed in the glomerulus of an MO patient. The fibril depositions are observed in the laminae rarae of the GBM and in the mesangium. **C** General morphology is disrupted due to the fibril deposition. In figure B, endothelial lumen (a) and fenestrated endothelial cells can be observed (c). The fibrils in the GBM appear to be collagenous in nature. c Scale bar = 5 µm

Evaluation of the renal sections by light microscopy did not reveal notable changes that could be specific for MO (Roberts and Gleadle 2008). A selection of representative images is shown in Fig. 3. However, EM analysis revealed a highly aberrant phenotype in one case with a noteworthy clinical history. This patient had received a kidney transplantation after suffering from a renal insufficiency. The primary disease was noted to have ‘membrane anomalies reminiscent of a hereditary nephritis’. We re-examined the EM grids of this patient, and found segmental fibrillar deposition that seemed similar to that reported by Roberts et al*.* (2008). Cross-striated fibrils with a diameter of 140 nm and regular periodicity were found in the mesangial area and the laminae rarae of the glomerular basement membrane of the native kidney (Fig. 4). This was the only patient in the cohort for whom EM data were available. Genetic data of this patient were not available.

## Discussion

In this study, we investigated the effect of heterozygous *EXT1* or *EXT2* germline mutations on glomerular permeability, glomerular morphology, and the endothelial glycocalyx. No (micro)albuminuria was observed in these patients, despite also having a higher prevalence of other risk factors for developing proteinuria with more hypertension, diabetes, and NSAID use, as shown in Table 1. Furthermore, no specific glomerular morphological changes were observed in biopsies from a historic cohort of MO patients without clinical symptoms of renal damage. However, one of the MO patients with renal pathology showed a glomerular phenotype resembling MO glomerulopathy. No significant difference was found in PBR thickness or vascular density between MO patients and controls.

This study shows that heterozygous *EXT1* or *EXT2* germline mutations do not significantly influence glomerular permeability or the endothelial glycocalyx. Glomerular permeability was not affected by these mutations, as no difference was found in albuminuria between patients and controls. We found no difference in PBR and vascular density between patients with an *EXT1* or *EXT2* mutation and controls, indicating that these mutations do not significantly alter the endothelial glycocalyx. Also, we found no specific glomerular phenotype in patients with osteochondromas. These results contribute to the widely discussed topic of the role of HS in the GFB (Chen et al. 2008; Khalil et al. 2019).

Interestingly, one patient in this group showed a complicated medical history with an unexplained renal insufficiency that resulted in renal replacement therapy. Our EM analysis of this patient showed glomerular deposition of large collagen fibrils in the mesangium and GBM, similar to those found by Roberts et al. (2008).

Previously, circulating HS derived from MO patients was found to be structurally normal, but quantitatively diminished compared to healthy controls (Anower et al. 2013). Whether MO patients have structurally aberrant HS in the GFB and glycocalyx is currently unknown. Our results could imply that the mono-allelic germline mutation in *EXT1* or *EXT2* in MO does not lead to these structural changes. Potentially, one functional allele is sufficient to prevent a phenotype from developing. Because most MO patients showed a normal glomerular morphology, we hypothesize that a second trigger is required to develop glomerular pathology specific to the *EXT1* or *EXT2* gene defect. In MO pathogenesis, germline heterozygous mutations in EXT1 or EXT2 are not sufficient to cause osteochondroma formation. Loss of heterozygosity is needed for osteochondroma to develop (Bovee et al. 1999; Reijnders et al. 2010). Analogous to this tenet, our data support the notion that a germline heterozygous mutation in the EXT1 or EXT2 gene alone is not enough to cause a pathologic phenotype in the glomerulus or endothelial glycocalyx. Cases, such as those described by Roberts et al*.* and here, are likely to have a distinct, local loss of heterozygosity and as such, loss of HS, leading to a specific phenotype. Potentially, factors other than HS could also play a role in these rare cases. Our group has previously shown that a bi-allelic germline mutation of EXT in zebrafish also does not lead to a renal phenotype, although it does result in a specific cartilaginous and dental phenotype (Khalil et al. 2019; Wiweger et al. 2011, 2012, 2014). Overall, based on the results from the current study and others, MO patients do not seem to have an increased risk of developing clinically significant microvascular complications. However, rare pathology exists in this disease and the current study might be underpowered to fully assess that risk.

A limitation of this study is that both glycocalyx measurements and urine samples were not collected multiple times. Glycocalyx measurements are of a dynamic nature with a small variability, and multiple measurements, as we have performed, increase reproducibility (Eickhoff et al. 2020).

Although multiple urine samples over a period of time would result in a more representative result of a patient’s typical albumin excretion, the absence of proteinuria, especially in the MO patient group, strongly suggests that indeed, no clinically significant pathology is present.

In this study, 6 out of 19 patients had a confirmed *EXT1* or *EXT2* mutation status. As stated in the introduction, the clinical diagnosis of the autosomal dominant disease of MO is often made without genetic testing, as in previous studies, a germline mutation in the *EXT1* or *EXT2* gene was found in almost 90% of cases (Bovee 2008; Hameetman et al. 2004). All MO patients in this study were included through the Dutch Multiple Hereditary Exostoses/Multiple Osteochondromas Association. In this study, outcome parameters were similar in subjects with and without a confirmed genetically confirmed diagnosis.

Most patients from the historic PALGA cohort did not have a confirmed *EXT1* or *EXT2* mutation. At least four patients from this cohort can be classified as having MO based on clinical criteria. The only clinical data we had available on these patients were the pathology reports. So this is most likely an underestimation of the total amount of MO patients within this cohort. We deduce that if a specific morphological phenotype with pathophysiological consequences would exist due to these mutations, our method results in an adequate appreciation of this topic. Besides the single patient described, the fact that no changes were found implies that *EXT1* or *EXT2* mutations do not necessarily cause changes to glomerular morphology. However, these results could be misinterpreted due to a lack of power.

In conclusion, this study shows that in humans, mono-allelic germline *EXT1* and *EXT2* mutations do not result in proteinuria or significant changes to the endothelial glycocalyx. Loss of heterozygosity could be an explanation for the very rare case of MO glomerulopathy described previously (Roberts and Gleadle 2008). Based on the current study, MO patients do not appear to be at an increased risk for clinically significant pathology of the glycocalyx or glomerular filtration barrier. Future studies might further elucidate the loss of heterozygosity theory and potentially identify other factors that might lead to the rare specific renal phenotype observed in only a very small subset of MO patients. This could assist in understanding not only the MO phenotype but also the pathophysiology of the glomerular filtration barrier and endothelial glycocalyx.

## Abbreviations

GAG: Glycosaminoglycan
GBM: Glomerular basement membrane
GFB: Glomerular filtration barrier
HME: Hereditary multiple exostoses
HS: Heparan sulfate
MO: Multiple osteochondromas
PBR: Perfused boundary region
RBC(w): Red blood cell (width)
